# Mechanisms of retinal ganglion cell injury following acute increases in intraocular pressure

**DOI:** 10.3389/fopht.2022.1007103

**Published:** 2022-12-09

**Authors:** Mary Anne Garner, Ryan G. Strickland, Christopher A. Girkin, Alecia K. Gross

**Affiliations:** ^1^ Department of Neurobiology, Heersink School of Medicine, University of Alabama at Birmingham, Birmingham, AL, United States; ^2^ Department of Ophthalmology and Visual Sciences, Heersink School of Medicine, University of Alabama at Birmingham, Birmingham, AL, United States

**Keywords:** retina, glaucoma, ocular hypertension, acute intraocular pressure (IOP), retinal ganglion cell

## Abstract

The maintenance of intraocular pressure (IOP) is critical to preserving the pristine optics required for vision. Disturbances in IOP can directly impact the optic nerve and retina, and inner retinal injury can occur following acute and chronic IOP elevation. There are a variety of animal models that have been developed to study the effects of acute and chronic elevation of IOP on the retina, retinal ganglion cell (RGC) morphology, intracellular signaling, gene expression changes, and survival. Acute IOP models induce injury that allows for the study of RGC response to well characterized injury and potential recovery. This review will focus on the initial impact of acute IOP elevation on RGC injury and recovery as these early responses may be the best targets for potential therapeutic interventions to promote RGC survival in glaucoma.

## Introduction

The loss of retinal ganglion cells (RGCs) in the retina and degeneration of the optic nerve is a hallmark of glaucomatous optic neuropathy, a leading cause for irreversible blindness worldwide (1, 2). Lowering of intraocular pressure (IOP) is currently the primary therapeutic intervention for glaucoma, though there are other possible causative factors that are responsible for RGC death in the disease (3, 4). In the last decade, several reviews have focused on the molecular mechanisms of (5–10) and neuroprotective strategies for (11–13) chronic glaucoma. While glaucoma is a heterogenous disease encompassing several different pathological phenotypes, here we review the morphological and molecular changes that RGCs and retinal glial cells undergo in models of acute increases in IOP.

Acute models of ocular hypertension vary in their execution, duration, and severity but generally produce a reversible loss of inner retinal function without inducing immediate RGC death. While the mechanism of RGC injury from either acute subischemic or ischemic injury may differ from the changes seen with chronic elevated IOP exposure, the acute IOP model can be performed with precisely controlled pressures and durations and provides a window into the mechanisms of RGC recovery. Additionally, electroretinography (ERG), used to measure retinal function, can be used as a metric for ensuring that the elevated IOP-induced injury remains subischemic since very high pressures can affect inner retinal function resulting in diminished *b*-waves (14).

There are numerous animal models of acute hypertension (15–34), and although nearly every model is different in mechanism, duration, and pressure elevation, we have included those that were evaluated within hours or days of pressure elevation unless otherwise explicitly stated. These models have resulted in an extensive body of knowledge regarding genes expressed and proteins up- or down-regulated. Throughout, we state whether the model discussed is acute or transient in nature and have added relevant findings from genetic and chronic models.

While the optic nerve and retinal structure vary across species, models of elevated IOP exhibit consistent patterns of RGC damage following injury. Though glaucoma is a chronic degenerative optic neuropathy, early disease stage signaling pathways and cell morphological changes are uncovered by studying the response of RGCs and their milieu to acute injury and the mechanisms by which they recover from induced damage (35).

## Organization of the retina

The neural retina is an organized tissue with distinct layers of neuronal and glial cells. The neuronal cell types include: 1) rod and cone photoreceptors, which transduce the energy of a photon of light into an electrical signal; 2) horizontal cells in the inner nuclear layer which synapse with photoreceptors; 3) bipolar cells, which receive input from photoreceptors and form synapses with RGCs; 4) amacrine cells in the inner plexiform layer form synapses with RGCs; and 5) RGC somas, dendrites, and axons. RGC axons form the optic nerve that projects to the lateral geniculate nucleus and suprachiasmatic nucleus of the thalamus (in primates) as well as to the superior colliculus of the brainstem and primary visual cortex within the occipital lobe (in primates and rodent models). The retina is also home to glial cells including astrocytes, Müller glia, and microglia. Each of these glial cell types play a role in RGC support, and each can subsequently become deleterious to RGC survival when activated for extended periods of time (e.g., following ocular hypertension).

Molecular profiling has indicated that there are approximately 40 subtypes of RGCs described in mice (36–39) and at least 18 different subtypes of RGCs in the primate retina (40, 41). Of the four major types studied in glaucomatous animal models (ON-sustained or transient and OFF-sustained or transient), the αOFF-RGCs, whose dendritic arbors form synapses with bipolar cells in the OFF sublamina of the inner retina, are the most vulnerable to morphological changes in dendritic arborization, synapse formation, and cell death following IOP elevation by laser (42) and microbead injection (43, 44). However, this effect may be due to age-related changes in dendritic morphology with OFF-RGCs being more adaptable and therefore able to recover more quickly following injury in younger animals (45). Additionally, the reported susceptibility of the larger αOFF-RGCs could be due to quantification of RGC subtypes based on soma size and an unintentional bias towards the loss of cells with larger somas if RGC cell bodies have shrunk in response to injury. ON- and OFF-sustained RGCs show evidence of normal dendritic arbors but loss of excitatory synapses within those arbors following microbead-induced IOP elevation (43), implying that changes in synapse formation may precede dendritic arbor morphological changes.

The mechanism of injury from acute IOP elevation is incompletely understood as elevation of IOP can impact the RCG axons directly and indirectly within the optic nerve head (ONH), and can alter inner retinal and ONH perfusion to the RGC soma and axons. In humans and higher primates, the axons of the RGCs pass through the lamina cribrosa before becoming myelinated and forming the optic nerve. Though rodent models lack the connective tissue found in human lamina cribrosae, they do possess a glial lamina containing astrocytes, and axoplasmic stasis can occur in the ONH of these models as well (46). There is an extensive body of work analyzing the effects of axonal damage on retrograde and anterograde transport and synapse degeneration, discussed below. The lamina cribrosa has been studied extensively as it is particularly susceptible to the damage induced by elevated IOP (47), resulting in axoplasmic stasis within the RGC axons. Remodeling of the lamina cribrosa in response to elevated IOP has been recently reviewed (48).

In addition to the axonal injury within the ONH region, there are also effects on the retinal vasculature and neural retina, especially in the inner retina, which can affect receptors in the dendritic membranes and calcium concentrations within the cells (49). Early gene expression and cell signaling changes that occur because of elevated IOP on the retina and on axonal transport are essential to understanding the early stages of glaucomatous damage (26).

## Models of ocular hypertension

RGC function, measured *via* ERG, is lost as a result of increases in IOP in animal models of experimental glaucoma while the outer retinal function is preserved (14, 16, 26, 50, 51). With acute models of very high IOP produced either transiently (17) or for a prolonged acute period (1 hour) (52), the functional recovery time for RGCs can be longer than that of the outer retina and may result in a lack of recovery even 4 weeks following insult (53). Transient increases in IOP are achieved through manometrically elevating IOP using a saline solution and are often used to produce pressures above that of the mean arterial pressure, producing an ischemic insult and preventing ocular perfusion. These high pressures not only affect RGC function but also result in loss of function of the outer retinal photoreceptors and bipolar cells. These studies often focus on functional recovery over time following insults of varying degrees. Studies have also indicated a relationship between blood pressure (BP), and therefore ocular perfusion pressure, and functional losses due to IOP (54).

Acute increases in IOP lead to nerve fiber layer thinning with a decrease in RGC soma number in rats (14, 52, 55) and mice (16), and degeneration of the optic nerve (18). Interestingly, there have also been reports of retinal nerve fiber layer thickening that is resolved by 3 weeks in rats exposed to 8 hours of IOP at 50 mmHg (56).

## Detecting elevation in IOP

How retinal cells initially respond to changes in IOP may be due to detection of mechanical stimuli *via* transient receptor potential (TRP) channels found throughout the retina (57) including in RGCs (58) and glia (59). This family of 28 cationic channels respond to mechanical stimuli such as strain or shear, inflammatory signals, changes in metabolic energy and oxidation (among other stimuli). An excellent recent review of TRP channels in all retinal cell types (60) discusses their role in retinal homeostasis and in response to injury, including their ability to induce astrocyte cytoskeletal reorganization and migration in response to elevated IOP (59, 61). Because TRP channels play a role in cellular homeostasis, their overactivation and inhibition can both result in gliosis. TRP channels remain a focus for investigation because their early sensing of mechanical changes following IOP may be what initially induce subcellular changes in retinal glia and RGCs.

## Morphological changes in RGCs: Axons and dendrites

### Axonal transport

**Figure 1:**
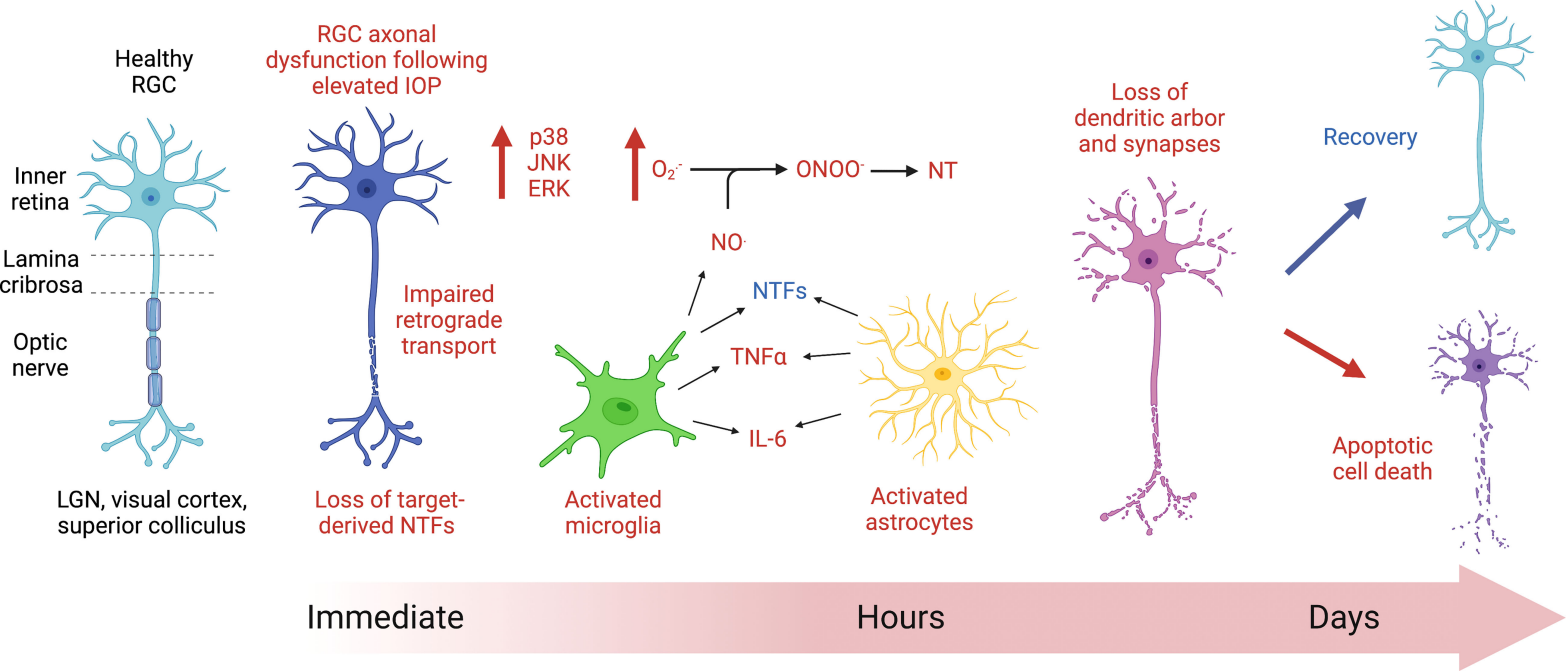
Cartoon diagram depicting the initial response of retinal ganglion cells (RGCs) to acute ocular hypertension. Axoplasmic stasis occurs immediately following RGC injury because of acute increases in intraocular pressure (IOP), affecting axonal transport. Within hours of injury, cell signaling changes occur, notably with the expression of mitogen activated protein kinases (MAPK, such as p38, JNK, and ERK). Glial cells, including microglia (*green*) and astrocytes (*yellow*), show evidence of increased reactivity within hours of the insult. Activated glial cells release nitric oxide (NO^.^), which, when combined with superoxide ( ${\text{O}}_{2}^{.-}$ ), can produce the highly reactive intermediate compound, peroxynitrite (ONOO^-^), which can then nitrate proteins on tyrosine residues (NT), interfering with protein function. There are noted morphological changes in RGC dendritic arborization within hours and throughout days following injury. Ultimately, the RGCs affected by increases in ocular hypertension will either recover or die via apoptosis.

One of the earliest morphological responses to elevated IOP is the interruption of axonal transport (**Figure 1**) (29, 30, 32, 62). Since neurotrophins can be target-derived and retrogradely transported along with their receptors in RGCs, the increase in IOP and subsequent axoplasmic stasis is detrimental to this vital process. The neurotrophin brain-derived neurotrophic factor (BDNF) and its receptor, TrkB, are normally trafficked retrogradely *via* dynein along axonal microtubules in RGCs. Retrograde transport of BDNF (22, 63) and TrkB (but not TrkA) are affected following acutely increased IOP in rats, similar to a chronic model of glaucoma in non-human primates (22). It is important to note that these studies detect effects in axonal transport hours following acute IOP elevation. When the analysis occurs one to two weeks following acute IOP elevation, there are no changes in anterograde or retrograde transport (56). This difference highlights the ability of RGCs to recover from acute models of hypertension. BDNF levels are notably low in glaucoma patients (5, 64, 65), and treatment with BDNF can be protective in glaucoma models (13, 66–68). BDNF protects RGCs exposed to increased IOP (69, 70) and is sufficient to increase proximal and distal axon density (71). However, the protections offered by BDNF are transient (72) and treatment with any neurotrophic factor would need to overcome the physical blood-retinal barrier as well as the limitation of neurotrophin half-life (73).

Mitochondria are also transported along axons in RGCs, and mitochondrial health is implicated in glaucoma since aged animals exhibit lower rates of transport and RGC dysfunction, resulting in the upregulation of Parkin, LC3-I and II, LAMP, and Optineurin (74). Effects on mitochondrial transport have been noted at 3 days following elevated IOP in a rodent model (75), though these trafficking defects may occur earlier as seen with BDNF and TrkB (22, 63). These early changes in axonal transport can precede RGC damage in acute and chronic disease models as axoplasmic statis has been shown to occur prior to RGC soma loss or presynaptic losses (**Figure 1**) (76). In an acute rat model of laser-induced ocular hypertension, axon transport deficits preceded RGC soma loss (77). In the chronic DBA/2J mouse model of ocular hypertension, anterograde transport is also affected early, even without exposure to increased IOP, and RGCs die *via* apoptosis (78).

### Dendritic morphology

Studies of IOP elevation in rats indicate initial increases in cell proliferation markers (79) and down-regulated pathways involved in axon extension, dendrite morphogenesis and metabolism of RGCs prior to the onset of apoptotic cell death (80). RGC degeneration involves dendritic changes including a loss of arborization and a decrease in synapse number. Dendritic branching and complexity can be assessed using a Sholl analysis where concentric circles are placed at regular intervals around the soma of the RGC and intersections are counted (81). Additionally, synapse number can be assessed using pre- and post-synaptic markers (e.g., synapsin, PSD95), and individual RGCs can be assessed using Thy-1 YFP transgenic mice where less than 1% of RGCs express the YFP, allowing for visualization of the entire dendritic arbor (81). Alterations in dendritic morphology occur prior to soma loss following acute IOP elevation in mice (82) as they do after optic nerve transection and chronic IOP elevation (81). In fact, dendritic morphological changes have been noted as soon as 90 minutes following acute elevation in IOP (82) (**Figure 1**). Various studies have investigated the early changes in RGC compartments after ocular hypertension (5, 6, 83–85). These changes include alterations in soma and dendrites as well as failures in axonal transport distally (83). How these changes relate to axonal injury at the nerve head or alterations in retinal perfusion remain unclear.

## Glial responses to acute ocular hypertension

Microarray studies in retina indicate an upregulation in inflammatory and immune pathways in the early stages of elevated IOP-induced damage. While these studies are retina-wide and do not distinguish among the cell types in which the changes occur, they provide evidence for changes in the early stages of glaucomatous damage. Early immune responses are detectable prior to RGC neurodegeneration, but whether these responses are protective or detrimental is unclear.

When neuronal damage occurs, glial inflammatory responses may initially produce a protective response, releasing neurotrophic factors and antioxidants, but prolonged reactivity of astrocytes and microglia can become excitotoxic to RGCs. Some acute models provide evidence for the upregulation of genes indicating activated microglia (*Aif1*) (79, 80) and reactive astrocytes and Müller glial (*Gfap*) (80), though others did not find changes in *Gfap* gene expression (79). There is also evidence for increased GFAP protein expression in rats (14, 86) and mice (16, 87) as early as 1 day following acute ocular hypertension (**Figure 1**). Glial reactivity and immune upregulation may therefore occur in early-to-mid states of glaucomatous disease (88), may not be upregulated uniformly across the retina or ONH, and may depend on the extent and duration of ocular hypertensive injury. GFAP protein expression is certainly increased in chronic models of ocular hypertension and in glaucomatous eyes (89).

Microglia are resident immune cells within the nervous system that respond to neuronal damage by proliferating and producing inflammatory cytokines such as tumor necrosis factor alpha (TNFα) (90). While initially neuroprotective (91), prolonged microglial activation can become detrimental. In glaucoma, microglial activity occurs during the early stages of the disease, prior to RGC apoptosis (91, 92), and treating DBA/2J mice with an inhibitor of microglial activation, minocycline, prevents some RGC apoptosis (93). Recent work has shown, however, that ablation of microglia causes significantly more RGC loss in a chronic microbead mouse model (94). The upregulation of heat shock proteins (HSP) by RGCs in response to elevated IOP can trigger microglial activation and inflammatory responses (95). HSPs are also elevated in human patients with glaucoma.

Acute elevation in IOP is sufficient to induce morphological changes in microglia in as little as one hour (**Figure 1**) (96), and activated microglia release pro-inflammatory molecules (e.g., complement proteins, nitric oxide (NO^.^), TNFα, interleukin-6 (IL-6), and FasL) which contribute to RGC damage and apoptotic cell death in glaucoma (97, 98). Cytokines such as IL6 and Lif have also been shown to be immediately upregulated in a rat model of acute IOP (79). Macrophages respond after acute IOP in rats (99) and mice (100) with increases in MCP-1, a macrophage chemokine, as early as 6 hours following acute IOP (101).

## Cell signaling pathways

Superoxide ( ${\text{O}}_{2}^{.-}$ ) is upregulated in the optic nerve and in the RGC somas within 24 hours (102), and free radical scavengers offer protection (103) in acute models. Superoxide and nitric oxide (NO^.^) can combine to form peroxynitrite (ONOO^-^) (**Figure 1**), which is a highly reactive negatively charged molecule that can nitrate tyrosine residues in proteins, altering their function. Nitrotyrosine levels have long been linked with inflammation and neurodegeneration and are elevated in primary open angle glaucoma (POAG) patient serum and in aqueous humor of animal models of glaucoma (104). The antioxidant ubiquinol (the reduced form of CoQ_10_) has been shown to be neuroprotective in one model of acute hypertension for at least 2 weeks following injury (105) as well as in the DBA/2J mouse model of ocular hypertension (106) by decreasing Bax expression and caspase-3 release, thereby preventing RGC apoptosis (105).

Increases in mitogen activated protein kinases (MAPKs) such as phosphorylated p38, JNK, and ERK are elevated within one hour following acute increases in IOP in the rat (106) as they are in glaucomatous eyes (98) (**Figure 1**). Inhibition of p38 MAPK has been shown to prevent degeneration of RGC axons in a microbead model of elevated IOP in rats while also protecting anterograde axonal transport (107–109). While JNK3 (110) and JNK2 (111) deficiencies are not protective, inhibition of JNK1/2/3 did spare RGCs exposed to acute high IOP (112).

## Discussion

Acute models of elevated IOP are used to elucidate the retinal response to this acute multifactorial insult. While chronic models of elevated IOP are able to analyze the fate of RGCs and their environment, acute models of IOP elevation provide a window into the initial subcellular and morphological changes that occur in RGCs and their neighboring retinal cells. The wide variety of models used to examine the effects of acute ocular hypertension on RGC morphology and cell signaling as well as glial reactivity have resulted in an enormous body of work across multiple animal species. Overall, they reveal the sensitivity of the inner retina to IOP related injury and the extent of the autoregulatory capacity of the inner retina, illuminating several critical pathways that mediate damage to the inner retina. While the mechanisms of acute IOP injury likely involve differing degrees of primary retinal ischemia when compared to chronic glaucoma, the use of acute models of ocular hypertension across several species is further strengthened by the fact that many of the pathways upregulated are also found in more prolonged models of ocular hypertension and in the later stages of chronic disease models as well as in human postmortem glaucomatous eyes. Uncovering the early responses of RGCs and their milieu to initial insults will be important in the search for therapeutic interventions in glaucoma since it is these initial changes that will likely be the optimal targets for pharmacological and/or genetic therapeutic interventions early in the disease, protecting RGCs from injury prior to irreversible loss. Expansion of acute models to include the human eye would further complement the work accomplished in animal models, providing translational validation for mechanisms of and treatments for the human disease.

## Author contributions

MG: Conceptualization, writing – original draft preparation, writing – review and editing, investigation. RS: writing – review and editing. CG: Conceptualization, review and editing, funding acquisition, supervision. AG: Conceptualization, writing – review and editing, funding acquisition, supervision. All authors contributed to the article and approved the submitted version.

## Funding

National Eye Institute: R01 EY0300096 (AG), National Eye Institute: R01 EY028284 (CG), EyeSight Foundation of Alabama (CG), Research to Prevent Blindness (CG).

## Acknowledgments

We would like to thank Dr. Lynn Dobrunz for invaluable insights for this work.

## Conflict of interest

The authors declare that the research was conducted in the absence of any commercial or financial relationships that could be construed as a potential conflict of interest.

## Publisher’s note

All claims expressed in this article are solely those of the authors and do not necessarily represent those of their affiliated organizations, or those of the publisher, the editors and the reviewers. Any product that may be evaluated in this article, or claim that may be made by its manufacturer, is not guaranteed or endorsed by the publisher.
